# Canonical WNT/β-Catenin Signaling Plays a Subordinate Role in Rhabdomyosarcomas

**DOI:** 10.3389/fped.2018.00378

**Published:** 2018-12-05

**Authors:** Nada Ragab, Florian Viehweger, Julia Bauer, Natalie Geyer, Mingya Yang, Anna Seils, Djeda Belharazem, Felix H. Brembeck, Hans-Ulrich Schildhaus, Alexander Marx, Heidi Hahn, Katja Simon-Keller

**Affiliations:** ^1^Institute of Pathology, University Medical Center Mannheim, University of Heidelberg, Mannheim, Germany; ^2^Institute of Pathology, University Medical Center Hamburg-Eppendorf, Hamburg, Germany; ^3^Institute of Human Genetics, University Medical Center Göttingen, Göttingen, Germany; ^4^Tumor Biology and Signal Transduction, Department of Hematology and Medical Oncology, University Medical Center Göttingen, Göttingen, Germany; ^5^Institute of Pathology, University Medical Center Göttingen, Göttingen, Germany

**Keywords:** rhabdomyosarcoma, canonical WNT signaling, myodifferentiation, WNT3A, FH535, XAV939

## Abstract

The development of skeletal muscle from immature precursors is partially driven by canonical WNT/β-catenin signaling. Rhabdomyosarcomas (RMS) are immature skeletal muscle-like, highly lethal cancers with a variably pronounced blockade of muscle differentiation. To investigate whether canonical β-catenin signaling in RMS is involved in differentiation and aggressiveness of RMS, we analyzed the effects of WNT3A and of a siRNA-mediated or pharmacologically induced β-catenin knock-down on proliferation, apoptosis and differentiation of embryonal and alveolar RMS cell lines. While the canonical WNT pathway was maintained in all cell lines as shown by WNT3A induced *AXIN* expression, more distal steps including transcriptional activation of its key target genes were consistently impaired. In addition, activation or inhibition of canonical WNT/β-catenin only moderately affected proliferation, apoptosis or myodifferentiation of the RMS tumor cells and a conditional knockout of β-catenin in RMS of *Ptch*^*del*/+^ mice did not alter RMS incidence or multiplicity. Together our data indicates a subordinary role of the canonical WNT/β-catenin signaling for RMS proliferation, apoptosis or differentiation and thus aggressiveness of this malignant childhood tumor.

## Introduction

Rhabdomyosarcoma (RMS) is a soft tissue malignancy with partially maintained skeletal muscle differentiation. RMS mainly affects children and young adults. Two main histological RMS subtypes are distinguished, embryonal RMS (ERMS) and alveolar RMS (ARMS). These subtypes show variable histological appearance, molecular characteristics and differ in outcome and progression of the disease (1, 2). ERMS account for 60–70% and ARMS for 30% of RMS. ARMS have a worse prognosis due to early metastasis formation (3). In contrast to the genetically imbalanced ERMS, tumors with an alveolar histology are mostly characterized by a reciprocal translocation of PAX3 or PAX7 and FOXO1. However, 25% of ARMS lack this translocation and are called “translocation negative” ARMS (4). Both ERMS and ARMS show features of skeletal muscle differentiation (5) with expression of *MYOD1*, myogenin *(MYOG)* and desmin *(DES)* (6). Nevertheless, RMS are poorly differentiated tumors with a differentiation status comparable to early myoblasts. Therefore, a mesenchymal stem cell as cell of origin is very likely (5).

Canonical WNT signaling is one of the key pathways regulating the differentiation of mesenchymal stem cells (7). The main control mechanism of this pathway is a constant degradation of β-catenin mediated by the so called “destruction complex” that is composed of different proteins including the scaffolding protein Axin, the adenomatous polyposis coli *(APC)* protein, casein kinase 1 *(CK1)* and the Ser/Thr kinase glycogen synthase 3 *(GSK3)*. In the absence of extracellular canonical WNT signaling, β-catenin is phosphorylated by GSK3 and subsequently degraded by the proteasome (8). Binding of WNT molecules to a receptor complex, consisting of the seven transmembrane protein frizzled *(FZD)* and the coreceptor low density lipoprotein receptor-related protein 5/6 *(LRP5/6)*, turns off β-catenin destruction by activating disheveled *(DVL)*, which in turn phosphorylates and deactivates GSK3. Thereby, phosphorylation of β-catenin is abolished, which leads to accumulation of β-catenin in the cytoplasm and translocation to the nucleus. β-catenin induces target gene expression by replacing the corepressor TLE at the transcriptional factor complex T cell factor/lymphoid enhancing factor 1 *(TCF/LEF1)* (9). Nowadays 19 human WNT proteins are known. Of these WNT1, WNT3A, and WNT7A determine the myogenic fate in the dermomyotome during development (10). During postnatal myogenesis and regeneration activation of the canonical WNT pathway is essential for differentiation and fusion of myoblasts into myotubes (11). In this context Annavarapou et al. reported on three observations in various ARMS and ERMS cell lines. First, recombinant WNT3A consistently elicited functional activity of the canonical WNT/β-catenin pathway through canonical pathway proteins. Second, WNT3A induced nuclear import of β-catenin followed by increased expression of *MYOG, MYOD1*, and *MYF5* and enhanced myodifferentiation. Third, myodifferentiation was accompanied by reduced proliferation of two ARMS cell lines but not of the two ERMS cell lines tested. Therefore, the authors assumed that canonical WNT signaling had a tumor suppressive role in some RMS tumors (12). However, these results are in contrast to our recently published data suggesting that it is the main interaction partner of β-catenin, LEF1, which suppresses aggressiveness and induces myodifferentiation of RMS cells, whereas β-catenin activity plays a subordinate role in these processes (13). In addition, a recently published paper by Bharathy et al. showed that activation of the canonical WNT signaling pathway in RMS in a patient derived xenograft model does not influence myodifferentiation or tumor progression (14). In the present work we try to unravel the impact of canonical WNT signaling and of β-catenin on aggressiveness and differentiation of RMS cells by (i) stimulation of RMS cell lines with WNT3A, (ii) β-catenin knockdown, (iii) using FH535, a small-molecule that inhibits β-catenin/TCF mediated transcription (15), and (iv) using XAV939 that antagonizes WNT signaling through AXIN stabilization and β-catenin degradation (16). We also conditionally knocked-out β-catenin in ERMS-like tumors in the mouse to investigate the role of β-catenin *in vivo*.

## Materials and Methods

### Cell Lines

The human alveolar RMS (ARMS) cell lines CRL-2061 (PAX3-FKHR translocation positive), the embryonal RMS (ERMS) cell lines RD and TE671 as well as the mouse fibroblast cell lines WNT3A (CRL-2647) and L cells (CRL-2648) were obtained from the American Type Culture Collection (ATCC, Menasses, VA, USA). The translocation negative ARMS cell line FLOH1 was a kind gift from Ewa Koscielniak, Stuttgart, Germany. CRL-2061 and FLOH1 were cultivated in RPMI1640 (Gibco, Carlsbad, CA, USA) with 10% (v/v) FCS (Sigma Aldrich, St. Louis, MO, USA). RD, TE671 and the mouse fibroblast cell lines WNT3A and L cells were maintained in DMEM (Gibco) with 10% (v/v) FCS. The conditioned medium with and without WNT3A was produced as described (ATCC). No antibiotics were added during cultivation. For all experimental settings cell cultures with similar cell densities were used to guarantee comparability of the results. These were for all cultivation conditions around 90%, since cell contacts are required to induce muscle differentiation.

All RMS tumor cells were sequenced by Sanger Sequencing to exclude potential activating β-catenin mutations.

### Antibodies and Chemicals

Two pharmacological inhibitors of the canonical ß-catenin pathway, FH535 and XAV939 were purchased from Sigma Aldrich and dissolved in DMSO (Sigma Aldrich) at a final concentration of 75 and 16 mM, respectively. IC_50_ of the drugs and the potential of the concentrations to inhibit β-catenin were tested by proliferation assays and western blots, respectively, in pilot experiments. The final concentration in cell culture experiments were 40 μM of FH535 and 16 μM of XAV939 if not indicate otherwise. DMSO (Sigma Aldrich) was used as vehicle control. For protein detection a monoclonal mouse anti-human β-catenin (Invitrogen, Carlsbad, CA, USA) and a mouse anti-human β-actin (Santa Cruz, Santa Cruz, CA, USA) antibody were used. For analysis of the subcellular localization, the following antibodies were used: monoclonal rabbit anti-human β-catenin (Cell Signaling Technology (CST), Danvers, MA, USA), non-phospho (active) monoclonal rabbit anti-human β-catenin (CST), monoclonal rabbit anti-human SP1 (CST), and monoclonal mouse anti-human GAPH.

Secondary antibody was a HRP-conjugated anti-mouse or anti-rabbit IgG (CST) for Western Blot. The presence of the fetal acetylcholine-receptor (fAChR) in RMS cell lines was detected by FACS using a mouse anti-human fAChR antibody (GeneTex, Irvine, CA, USA) and an Alexa Fluor 488-conjugated goat anti-mouse IgG (Jackson ImmunoResearch, West Grove, PA, USA). The knock-down of ß-catenin was performed with siRNA (SI02662478, Qiagen). As control AllStars negative control siRNA (SI03650318, Qiagen) was used. Transfections of the siRNAs were performed with HiPerFect (Qiagen, Hilden, Germany) according to the manufacturer's instructions. Recombinant human WNT3A was purchased from R&D Systems (Minneapolis, MN, USA) and used at a final concentration of 200 ng/ml. The protein is produced in a CHO cell line to guarantee the correct posttranslational modification for WNT3A.

Analysis of RMS mouse tumors were done by using a rabbit anti-mouse β-catenin antibody, a rabbit anti-mouse BCL9-2, a rabbit anti-mouse BCL9, and a rabbit anti-mouse LEF1 antibody as described (17).

### Cytotoxicity Assay

Cytotoxicity was monitored by an MTT (Thiazolyl blue TetrazoliumBromid)-based colorimetric assay (Sigma Aldrich). RMS cells were treated with either FH535, XAV939, siRNA, WNT3A, or L cell conditioned medium, in round-bottom 96-well-microtiter plates. After 0, 24, 48, and 72 h, 20 μl MTT (5 mg/ml) was added and cells were incubated for another 3 h at 37°C. Before measurement supernatant was discarded and MTT crystals were dissolved in 200 μl DMSO. Reduction of MTT by viable tumor cells was colorimetrically determined by a microtiter-plate-reader (Tecan, Männerdorf, Switzerland) at 565 nm and a reference wavelength of 670 nm. Three independent experiments were performed, each in triplicate. Data were analyzed with Student's *t*test using the Software tool GraphPad Prism version 7.0 for Windows (GraphPad Software, La Jolla California USA, www.graphpad.com).

### Migration Assay

For the migration test (“scratch assay”) cells were cultivated in a 12-well plate (0.5 × 10^5^ cells/well) and pretreated with FH535, XAV939, WNT3A, or L cell conditioned medium for 24 h. When confluence was reached, two scratches, which were vertical to each other, were made with a pipette tip per well. To remove detached cells, the cells were washed once before adding FH535, XAV939, WNT3A, or L cell conditioned medium. After 0, 4, 8, 12, and 24 h, photos of the scratches were taken at the inverse microscope with phase contrast at 100 × magnifications (Leica, Wetzlar, Germany). For analyzing the photos, the ImageJ plugin Scratch Assay Analyzer was used. Three independent experiments were performed.

### Real Time PCR

Total RNA was extracted by using TRIzol reagent according to the manufacturer's instructions (Invitrogen). Reverse transcription PCR (RT-PCR) was performed using “RevertAid H Minus First Strand cDNA Synthesis Kit” (Thermo Fisher Scientific; Waltham, MA, USA). qRT-PCR was done by using the “Step one plus system” (ABI, Thermo Fisher Scientific). All primers are listed in Supplemental Table 1. The amplification products were detected with “DyNAmo ColorFlash SYBR Green qPCR Kit” (Thermo Scientific). The ΔΔCT-method (18) was used for quantification and Student's *t*test for statistical analysis (GraphPad Prism version 7.00 for Windows). Expression levels of the target mRNAs were normalized to GAPDH mRNA.

### Western Blotting

Protein extraction was done by using TRIzol reagent or the NE-PER Nuclear and cytoplasmic extraction kit according to the manufacturer's instructions (Invitrogen, Carlsbad, CA, USA). Proteins were separated on 10% SDS-Polyacrylamid gels and were transferred to a polyvinylidene fluoride-membrane (PVDF, GE Healthcare, Fairfield, CT, USA). Membranes were blocked in 5% low fat milk for at least 1 h at room temperature or overnight at 4°C. Antibodies were diluted 1:1,000 in TBST, incubated overnight at 4°C (primary antibody) or for 1 h at room temperature (secondary antibody). The proteins were visualized with ECL Plus Substrat (Pierce, Thermo Scientific) and the Fusion SL Imaging system (Peqlab, Erlangen, Germany). Protein expression was analyzed with the Fusion-Capt-Software (Peqlab). Densitometric quantification was done with ImageJ (19).

### Flow Cytometry Assay

For flow cytometry analysis 2 × 10^5^ cells/well were seeded in a 6-well plate. After attachment, cells were treated for 48 h with L cell (control) or WNT3A conditioned medium. For flow cytometric analysis cells were harvested and washed three times with ice cold PBS. Cells were incubated with a primary anti-fAChR antibody (1:100) on ice for 1 h and washed three times before adding the secondary detection antibody and subsequent incubation for 15 min on ice. Before measurement cells were washed again three times with ice cold PBS and analyzed in 200 μl ice cold PBS by using a FACSCanto™ II (BD Bioscience).

For detection of apoptosis, RMS cells were treated with either FH535, L cell, or WNT3A conditioned medium for 48 h and stained with Annexin V/Propidium iodide using the Annexin V Apoptosis Detection Kit from BioLegend (San Diego, CA, USA) according to the manufacturer's instruction. Measurements were done by using the FACSCanto™ II (BD Bioscience) and analyzed with FACSDiva™-software (BD Bioscience).

For the BrdU (proliferation) assay cells were pretreated for 4 h with FH535 (40 μM), XAV939 (16 μM) or WNT3A and BrdU was added for the last 24 h. BrdU staining and flow cytometry analysis were done according to the manufacturer's instructions (BD Bioscience).

### Sphere Assay

Cells were pre-treated for 48 h with 20 μM FH535 and 16 μM XAV939 or with 200 ng/ml rWNT3A. For long term cultivation experiments, cells were pretreated for 96 h with L cell conditioned media, WNT3A conditioned media or media containing 200 ng/ml rWNT3A.

Rhabdomyosarcoma sphere cultivation was done as described by Walter et al. (20). Sphere media was freshly prepared and contained the following components: Neurobasal medium (Life technologies), 10 ng/ml EGF (R&D Systems), 20 ng/ml b-FGF (R&D Systems), and 2 × B27 (Life technologies). 2 cells/μl media were applied and seeded out in ultra-low attachment plates (Sigma Aldrich). For a permanent treatment, inhibitors and rWNT3A were directly added to the sphere media at the following concentrations: 5 μM FH535, 16 μM XAV939, and 200 ng/ml rWNT3A.

### Genetic RMS Models

Experiments using animals were performed with consideration of all necessary legal requirements and have been approved by the Lower Saxony State Office for Consumer Protection and Food Safety (file number 33.9-42502-04-12/0805). Because *Myf5* is expressed in RMS of *Ptch*^*del*/+^ mice (21, 22) (see below), ß*-cat*^*flox*/*flox*^ (23) mice on a C57BL/6 background were crossed with *Myf5*^*CreER*^ mice on a Balb/cJ background. *Myf5*^*CreER*/*CreER*^ mice express a tamoxifen-inducible Cre recombinase within the 3′ untranslated region of the *Myf5* gene following the stop codon in exon 3 (24). In parallel, ß*-cat*^*flox*/*flox*^ mice were also bred to *Ptch*^*del*/+^ mice that harbor a heterozygous *Ptch* germline mutation [for generation of *Ptch*^*del*/+^ from *Ptch*^*flox*/*flox*^ mice see (25)]. *Ptch*^*del*/+^ mice were on a pure Balb/cJ background that confers high RMS susceptibility (22, 26). Resulting β*-cat*^*flox*/+^*Myf5*^*CreER*/*wt*^ mice were again crossed to *Myf5*^*CreER*/*CreER*^ mice, whereas β*-cat*^*flox*/+^*Ptch*^*del*/+^ were crossed to ß*-cat*^*flox*/*flox*^ mice. β*-cat*^*flox*/+^*Myf5*^*CreER*/*CreER*^ mice were crossed to the resulting β*-cat*^*flox*/*flox*^*Ptch*^*del*/+^ mice to obtain β*-cat*^*flox*/*flox*^*Ptch*^*del*/+^*Myf5*^*CreER*/*wt*^ mice. Latter mice were injected with 1 mg tamoxifen (10 mg/ml in sterile ethanol:sun flower oil, 1:10) intraperitoneally (i.p.) on five consecutive days (cumulative dose 5 mg) at an age of 4 weeks. Uninjected or solvent injected β*-cat*^*flox*/*flox*^*Ptch*^*del*/+^*Myf5*^*CreER*/*wt*^ mice served as controls. Additionally, we also used β*-cat*^*flox*/*flox*^*Ptch*^*del*/+^ for the experiments to exclude potential leakiness of the cre-driver and tamoxifen-mediated effects on RMS growth.

Mice were monitored for tumor formation weekly. If possible, the observation period encompassed at least 200 days. Mice were sacrified after the observation period and all mice were examined for additional, non-visible and non-palpable tumors. The identity of the tumors as RMS was confirmed using paraffin sections stained with hematoxylin and eosin (HE).

All primers used for genotyping are listed in Supplemental Table 1. Genotypings are also described in Uhmann et al. (27), Zibat et al. (25), Huelsken et al. (23), Yu et al. (28), and Biressi et al. (24). In addition, the degree of recombination at the targeted *Lef1* or β*-cat* locus in DNA isolated from RMS and normal skeletal muscle was estimated by PCR (for primers see Supplemental Table 1).

### Statistical Analysis

Data were analyzed with Student's *t*test using the Software tool GraphPad Prism version 7.00 for Windows (GraphPad Software, La Jolla California USA, www.graphpad.com).

## Results

### Downregulation of β-Catenin Expression by FH535, XAV939, and β-Catenin Specific siRNA

**Figure 1:**
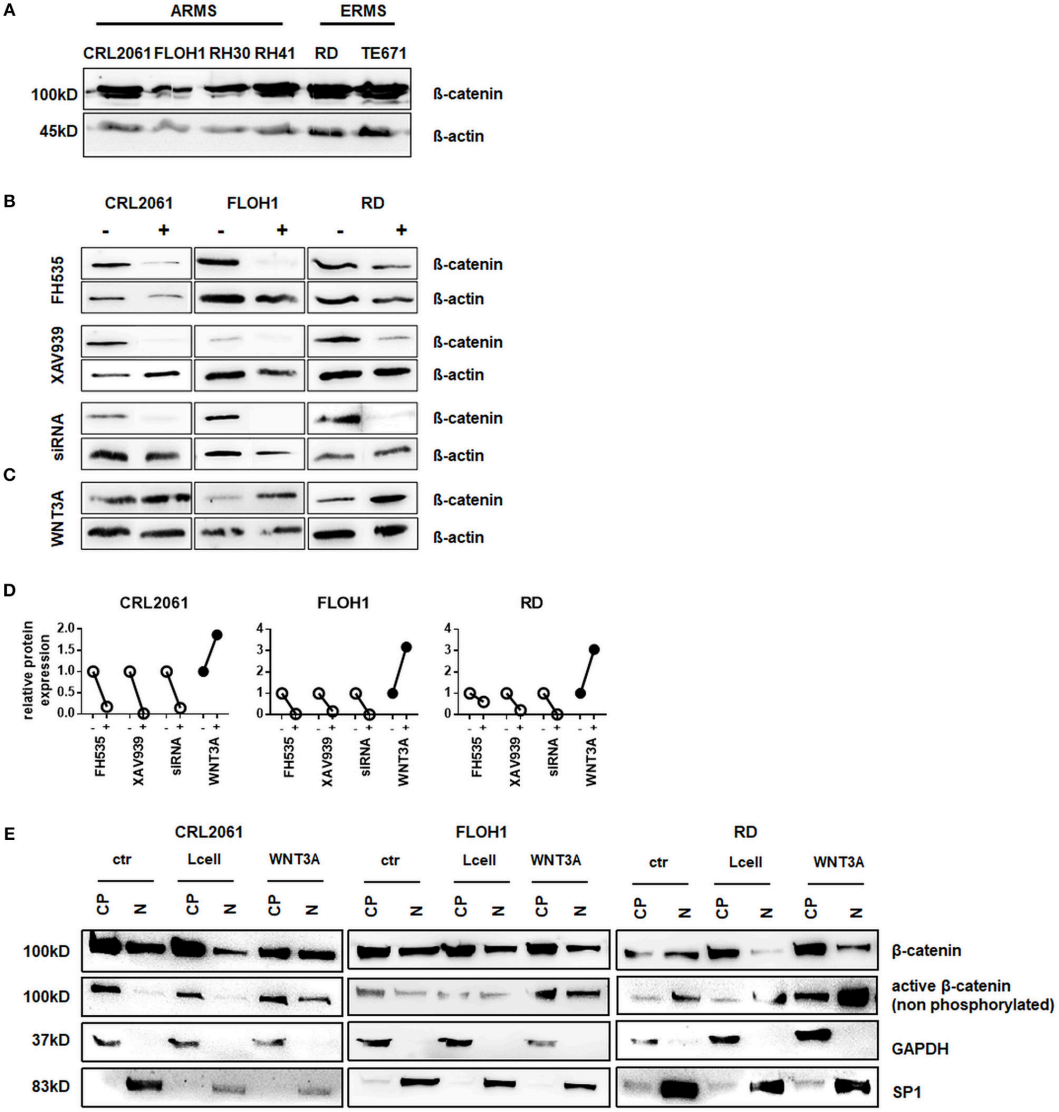
Western Blot analysis of β-catenin expression in RMS tumor cell lines. **(A)** Western Blot analysis of ß-catenin expression in various RMS tumor cell lines. ß-actin served as loading control. **(B)** Western Blot analysis of ß-catenin expression in three different RMS tumor cell lines after 48 h treatment with either FH535, XAV939, ß-catenin siRNA, or **(C)** WNT3A treatment. ß-actin served as loading control. **(D)** Densitometric quantification of the Western Blots shown in **(C)** using ImageJ. Expression of ß-catenin after treatment (+) was normalized to ß-actin and compared to untreated cells (−). **(E)** subcellular localization of ß-catenin in the RMS tumor cell lines with and without WNT3A stimulation. GAPDH served as cytoplasmatic and SP1 as nuclear marker to check purity of the protein isolation from different compartments. CP, cytoplasmatic; N, nuclear.

We and others showed that β-catenin is expressed in various RMS cell lines (Figure 1A) and tissue (13). As pre-requisite to evaluate the functional relevance of the canonical WNT pathway for RMS (see below), β-catenin was either inhibited or induced in three RMS cell lines that represent the major RMS subtypes. These were PAX3 translocation positive CRL2061 ARMS cells, translocation negative FLOH1 ARMS cells and RD ERMS cells. The canonical pathway was blocked by using FH535 and XAV939. Both inhibitors reduced β-catenin protein levels in all cell lines as did transfection with β-catenin-specific siRNA (Figure 1B). In contrast, addition of WNT3A conditioned medium or rWNT3A to RMS cell lines increased the amount of cellular β-catenin due to activation of the canonical WNT signaling pathway and stabilization of the β-catenin protein (Figures 1C,D and Supplemental Figure 1A). An analysis of the subcellular localization of β-catenin revealed cytoplasmatic and nuclear localization in all three tested RMS cell lines, which was independent of the respective treatments (Supplemental Figure 2). In addition, we found an increased amount of nuclear active (non-phosphorylated) β-catenin after WNT3A treatment (Figure 1E).

### β-Catenin Does Not Influence Proliferation/Cell Growth and Migration of RMS Tumor Cells

Activation of the canonical WNT pathway has been described to affect proliferation and migration of various non-rhabdomyomatous tumor cells through induction of the targets such as the pro-proliferative protein *MYC* and the pro-proliferative and anti-apoptotic IAP-protein *BIRC5* (survivin) (29, 30). We here tested the effects of FH535, XAV939, β-catenin siRNA, and WNT3A treatment on proliferation and expression of the mentioned β-catenin target genes in the above mentioned RMS cell lines.

**Figure 2:**
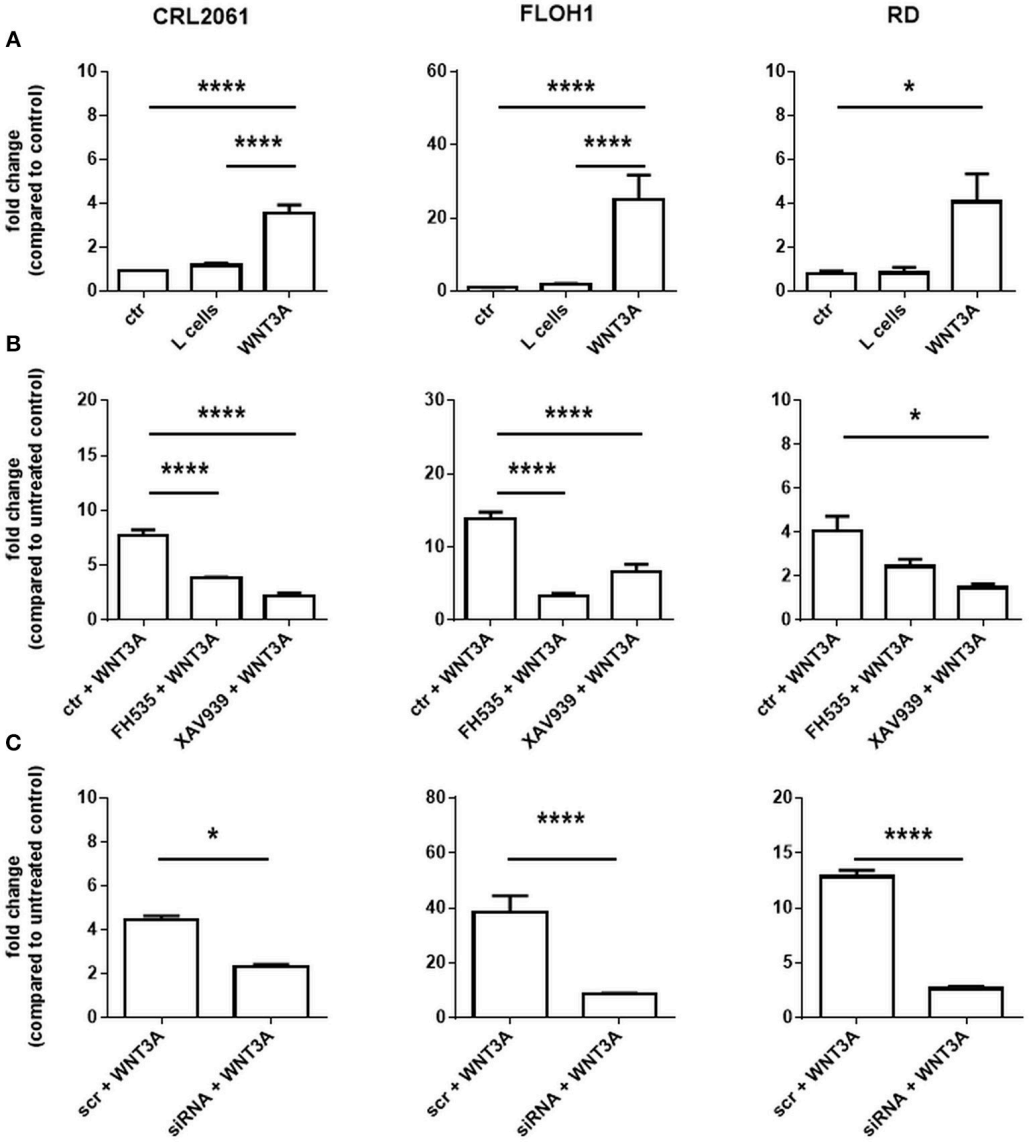
Effects of FH535, XAV939, ß-catenin siRNA, and/or WNT3A on the expression level of the ß-catenin target gene *AXIN2* in RMS cell lines. **(A)** *AXIN2*-specific qRT-PCR without or after 24 h of pretreatment with FH535, XAV939 **(B)** or ß-catenin specific siRNA **(C)**. Please not that the batches of WNT3A conditioned medium were not identical. *GAPDH* expression was used for normalization. Shown is the mean and SEM of three independent experiments measured in duplicates; ctr, control; scr, scrambled RNA; significance level: ^*^p < 0.05, ^****^p < 0.0001.

First, we measured the expression of *AXIN2*, which is a typical response gene of WNT3A. As shown in Figure 2A *AXIN2* was significantly upregulated in all three cell lines upon treatment with WNT3A (Figure 2A) and this upregulation was significantly suppressed when the cells were additionally incubated with FH535, XAV939, or ß-catenin-specific siRNA (Figures 2B,C). These results show that, as expected, WNT3A induces the expression of *AXIN2* in RMS cell lines, which apparently is dependent on ß-catenin.

**Figure 3:**
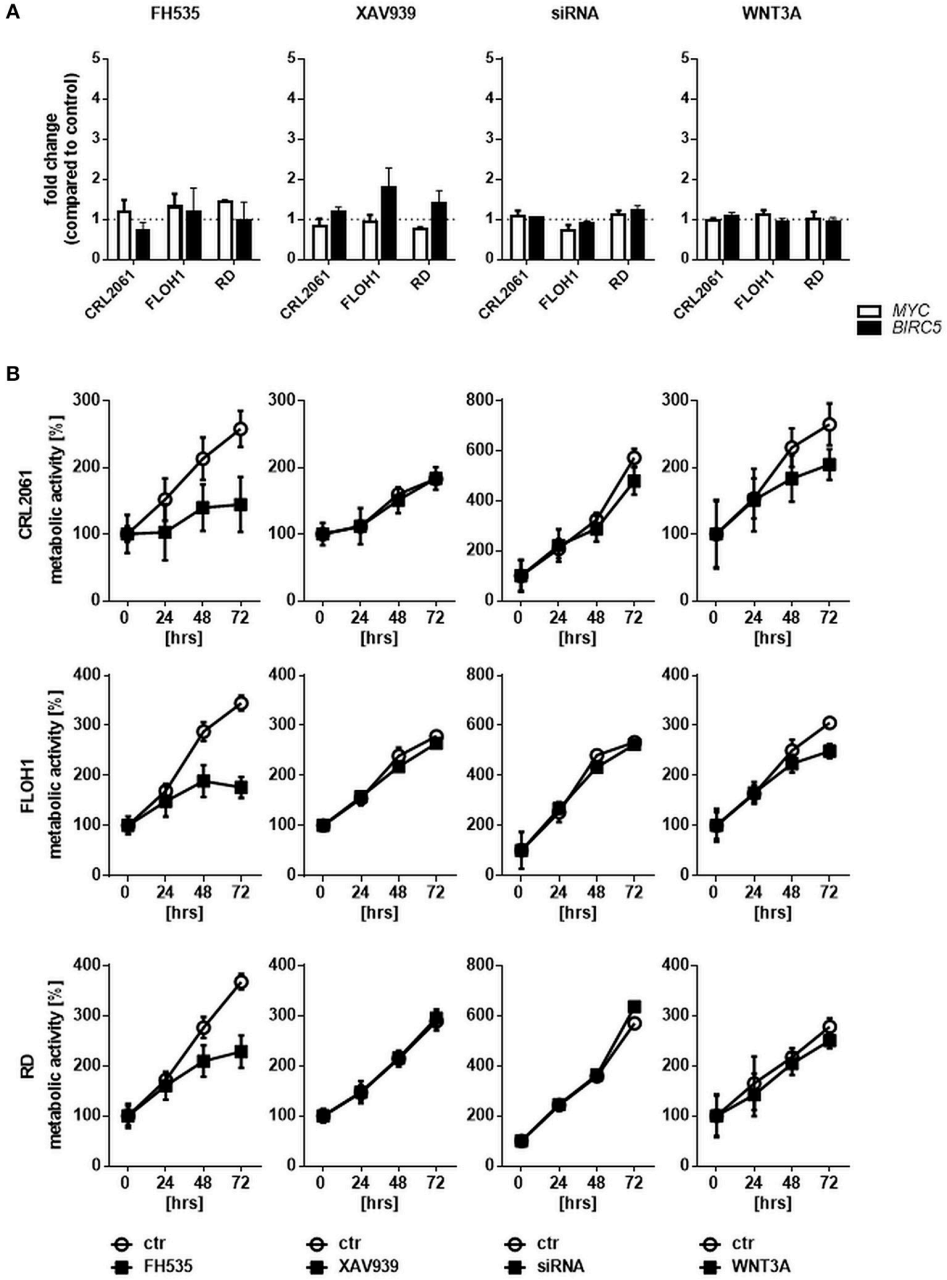
Effects of FH535, XAV939, ß-catenin siRNA, or WNT3A on proliferation of RMS cell lines. **(A)** qRT-PCR of the ß-catenin-target genes *MYC* (white bars) and *BIRC5* (black bars) after incubation with FH535 (40 μM), XAV939 (16 μM), ß-catenin siRNA, or WNT3A conditioned medium for 48 h. *GAPDH* was used for normalization. Shown is the mean and SEM of three independent experiments measured in duplicates and compared to the solvent control. **(B)** represents MTT test to examine the influence of 40 μM FH535, 16 μM XAV939, ß-catenin siRNA, or conditioned WNT3A Medium on metabolic activity of the ARMS cell lines CRL2061 and FLOH1 and the ERMS cell line RD over 72 h. DMSO and scrambled siRNA served as control (ctr). The graphs show one out of three experiments, measured in triplicates.

Next, we measured the expression of the β-catenin target genes *MYC* and *BIRC5*. As shown in Figure 3A, activation of WNT3A signaling did not influence the expression in any of the three cell lines. In addition, we did not see any effect when the cells were incubated with ß-catenin-specific siRNA or the ß-catenin inhibitors FH535 or XAV939. These data indicate that the ß-catenin signaling axis apparently is not involved in induction of *MYC* and *BIRC5* in RMS cell lines. In addition, we analyzed the effect of FH535, XAV939, ß-catenin-specific siRNA, and WNT3A on metabolic activity and cellular proliferation. Metabolic activity of all RMS cell lines as measured with the MTT assay was unchanged after XAV939 or β-catenin siRNA treatment. In contrast, FH535 significantly reduced the metabolic activity of CRL2061 cells after 24 h and that of FLOH1 and RD cells after 48 h (Figure 3B).

Paradoxically, WNT3A conditioned medium reduced the metabolic activity of the ARMS cell lines CRL2061 and FLOH1 in comparison to the control (Figure 3B, right panel). This was predominantly seen after 72 h of treatment. However, when rWNT3A was used in the same experimental setting metabolic activity of the cells was not affected, although *AXIN2* expression was induced as expected (Supplemental Figure 1). These results, together with those of the BrdU incorporation assay (see below), implicate that the deceleration of cellular growth was related to the already consumed medium (see also Materials and Methods section for the collection method of WNT3A medium).

**Figure 4:**
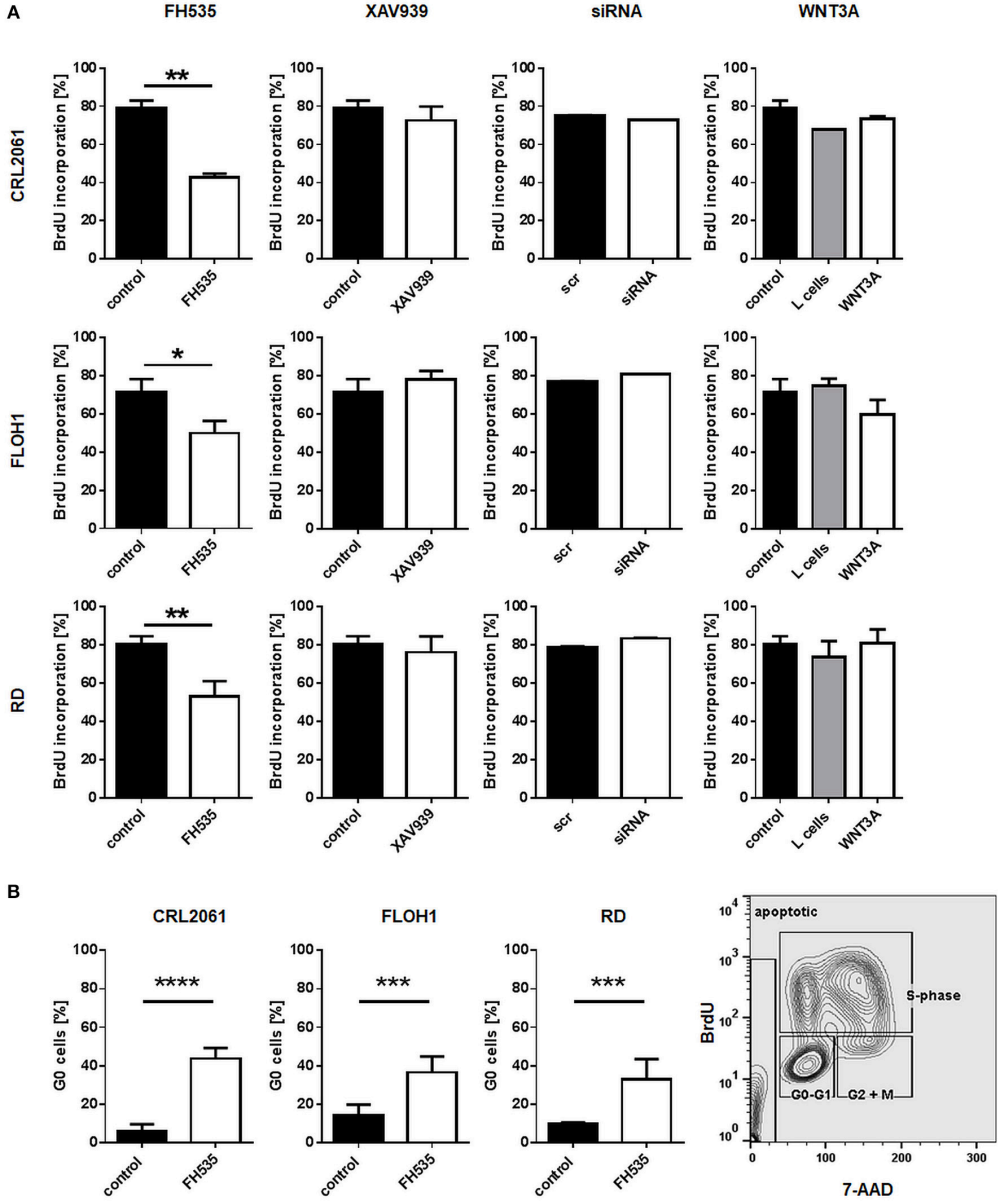
Effects of FH535, XAV939, ß-catenin siRNA, or WNT3A on proliferation of RMS cell lines. **(A)** Incorporation of BrdU after treatment of RMS tumor cells with FH535, XAV939, a ß-catenin-specific siRNA, or WNT3A conditioned medium. The mean percentage and SEM of BrdU positive cells out of three independent experiments are shown. **(B)**. Analysis of G0 cells with and without FH535 treatment. The bars represent mean percentage and SEM of G0 cells of three independent experiments. Right graph shows the gating strategy on the basis of FH535 treated RD cells. Significance level: ^*^p < 0.05, ^**^p < 0.01, ^***^p < 0.001, ^****^p < 0.0001.

To further analyze the effects of FH535, XAV939, ß-catenin, and WNT3A conditioned medium a BrdU test was performed. Similar to the MTT test, FH535 treatment inhibited proliferation after 24 h, whereas the other treatments did not (Figure 4A). FH535-mediated inhibition in all likelihood was due to an arrest of the cells in G0 as shown by FACS analysis (Figure 4B).

**Figure 5:**
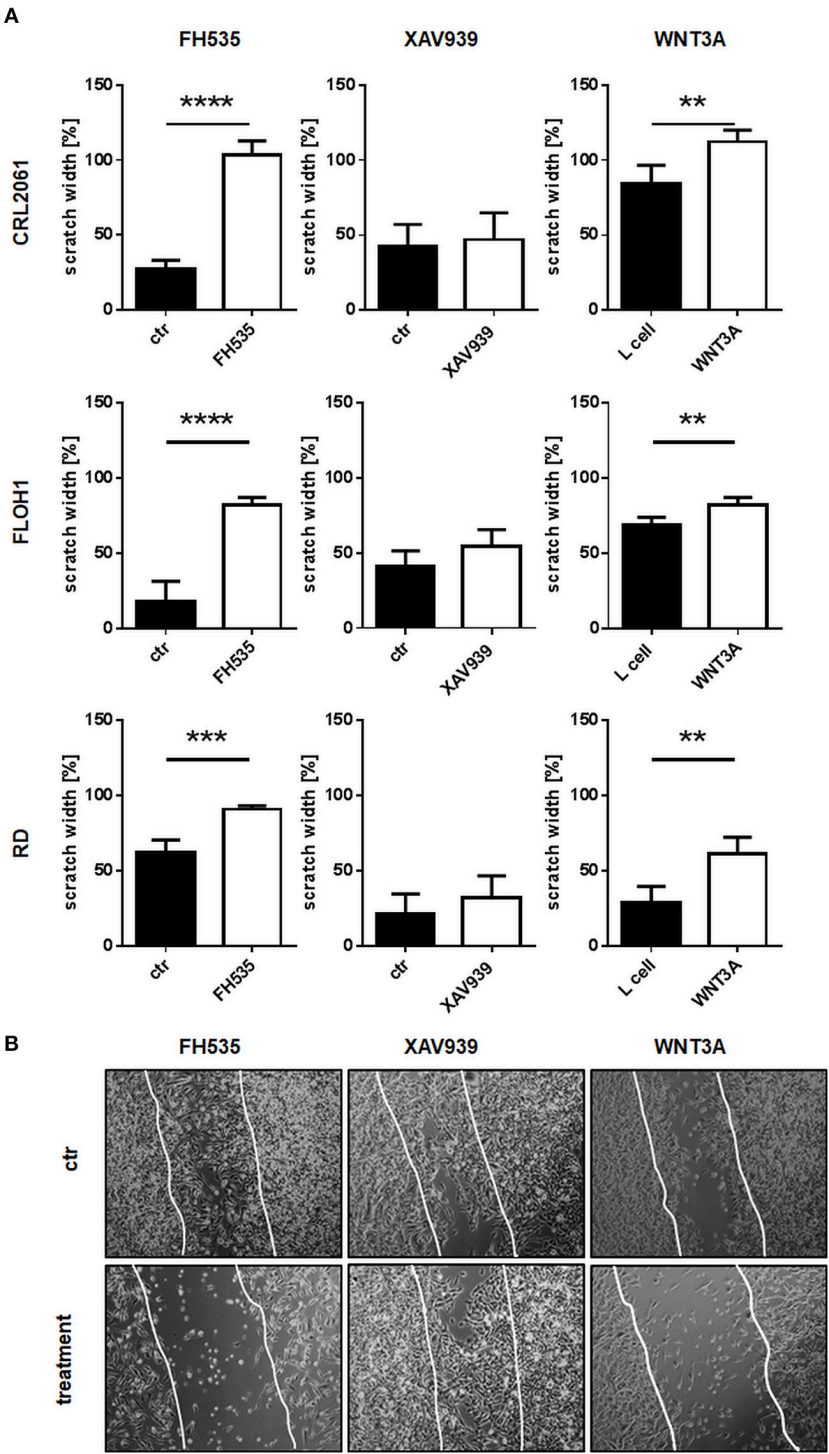
Effects of FH535, XAV939, ß-catenin siRNA, or WNT3A on migration of RMS cell lines. **(A)** RMS tumor cells were pre-treated with FH535, XAV939, or WNT3A for 24 h before making the scratch. Scratch width of untreated controls at time zero was set to 100%. After 0, 4, 8, 12, and 24 h scratch width was measured at four different positions. The graphs show the mean and SEM after 24 h treatment. **(B)** Pictures of the scratch in control and treated RD cells after 24 h using phase contrast with a magnification of 100x. Significance level: ^**^p < 0.01, ^***^p < 0.001, ^****^p < 0.0001.

Next a scratch test was performed to check if ß-catenin inhibitors or WNT3A influence the migratory abilities of the tumor cells. To achieve similar experimental conditions as described for the proliferation and differentiation assays (see above), cells were pre-incubated with inhibitors or conditioned medium for 24 h. In accordance with the former results and besides inhibition of proliferation, FH535 treatment induced a migration arrest, whereas cells treated with XAV939 showed a control-like behavior. In contrast, treatment with WNT3A conditioned medium slightly reduced migration of all RMS cell lines (Figure 5A; original pictures are shown in Figure 5B).

**Figure 6:**
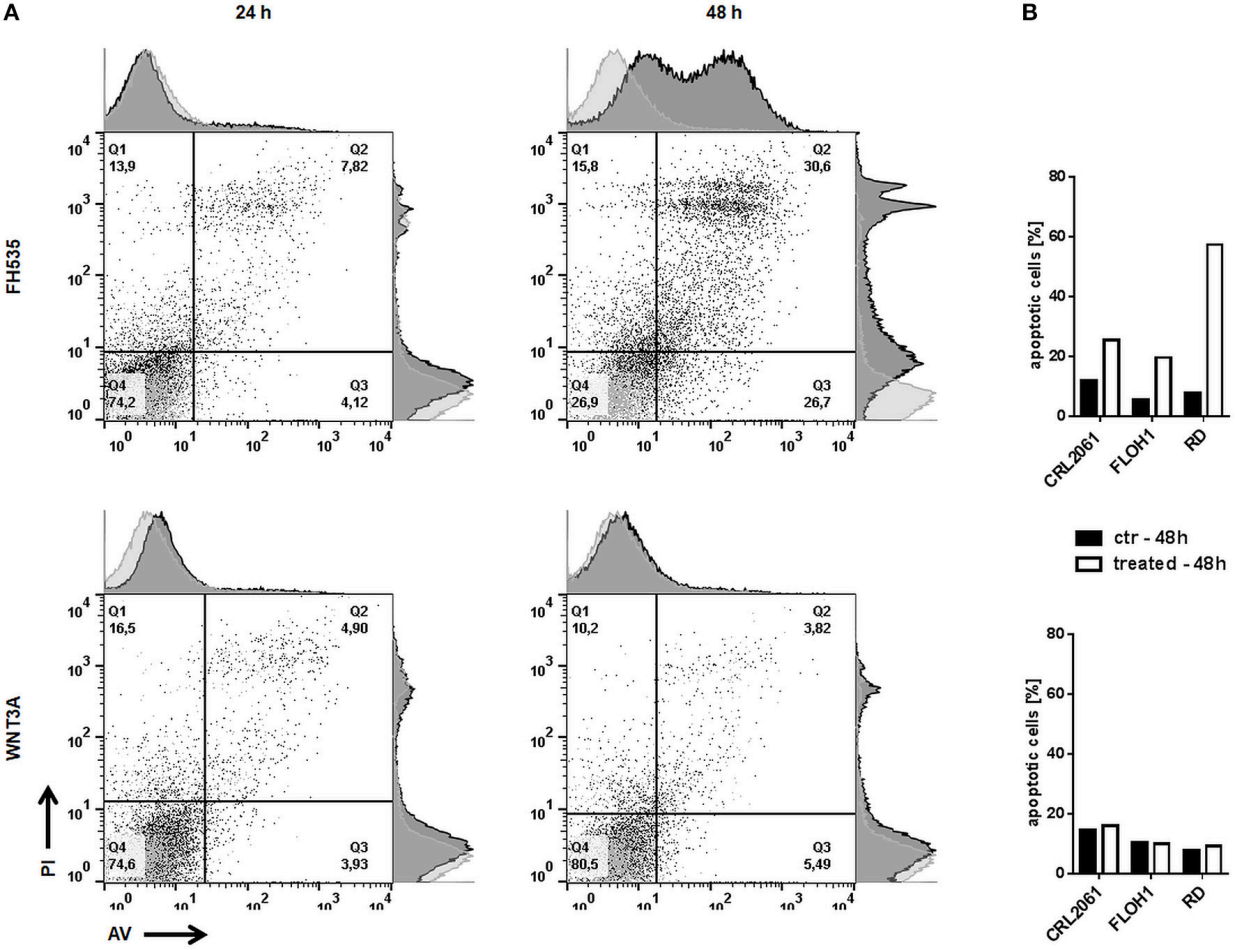
Effects of FH535 and WNT3A on apoptosis of RMS cell lines. **(A)** Flow cytometry analysis of Annexin V/PI positive RD tumor cells 24 and 48 h after treatment with FH535 (upper panel) or WNT3A (lower panel). An additional histogram plot on the Annexin V- (x-) axis and PI- (y-) axis illustrates the shift of apoptotic cells (dark gray) compared to untreated control cells (light gray). **(B)** represents a graphic summary of the apoptotic cells of all three cell lines in percent after 48 h of treatment.

Since FH535 and WNT3A treatment apparently altered the growth behavior of the cells, we also measured apoptosis after 24 and 48 h of incubation. The amount of apoptotic (Annexin V/PI double positive as well as Annexin V single positive) cells did not increase after WNT3A treatment. However, FH535 strongly induced apoptosis in all RMS cell lines, especially in RD, after 48 h (Figure 6).

Next, we tried to unravel what causes the difference in cellular growth behavior when cells are incubated with FH535 that inhibits β-catenin/TCF mediated transcription compared to XAV939 that antagonizes WNT signaling through β-catenin. Since it is known that FH535 not only affects target gene expression of the canonical WNT pathway, but also inhibits the expression of the transcription factors *PPARG* and *PPARD* (15), we analyzed these proteins by means of Western Blot. Indeed, *PPARG* was reduced on protein level after FH535 treatment (Supplemental Figure 3) whereas treatment with XAV939 or specific β-catenin siRNA had no effect. Therefore, the FH535-mediated effects may not dependent on β-catenin, but on regulation of PPAR proteins.

### Neither Activation nor Inactivation of the Canonical WNT Pathway Modulate the Differentiation Status of RMS Cell Lines

**Figure 7:**
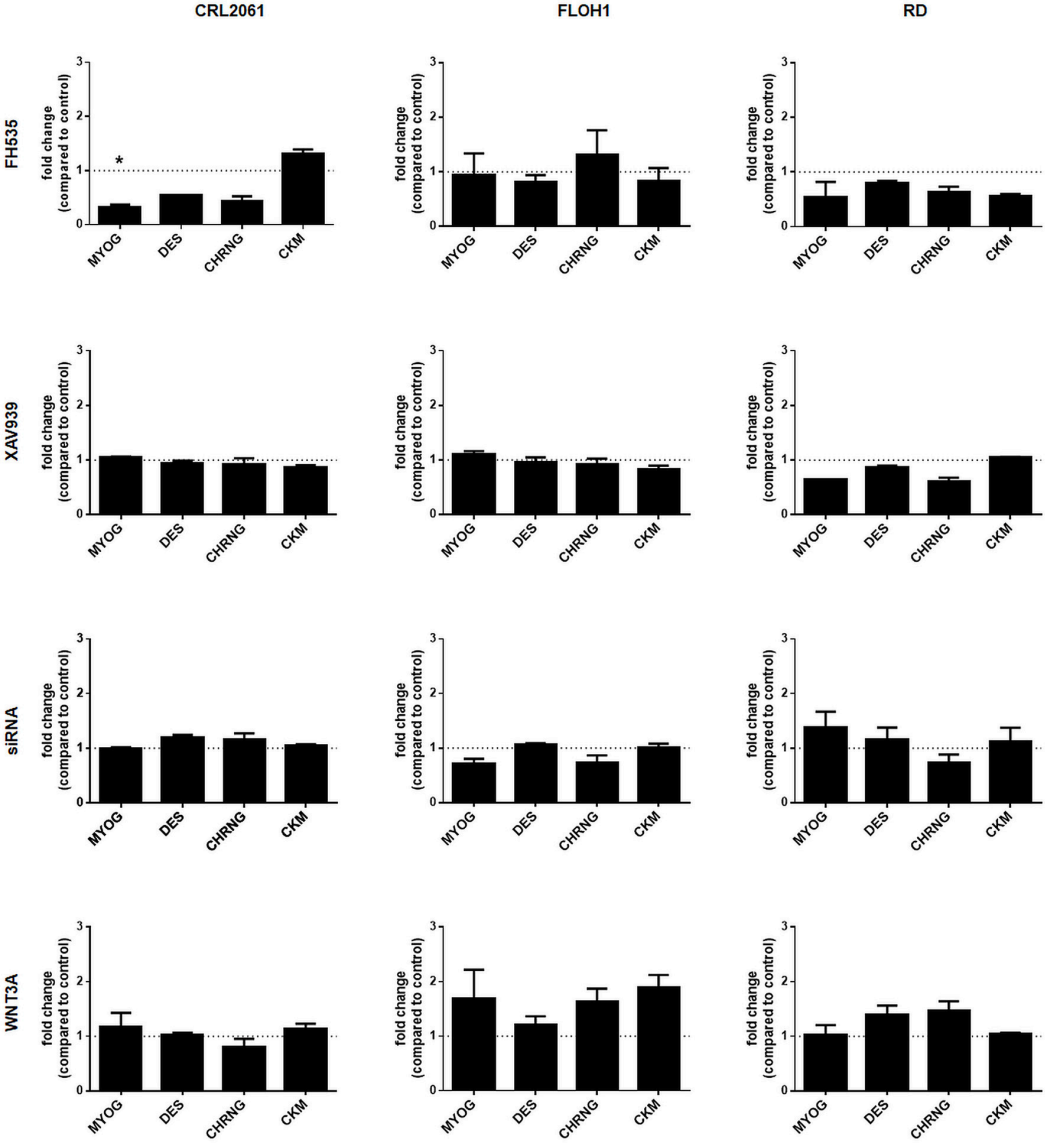
Effects of FH535, XAV939, ß-catenin-siRNA, or WNT3A on the expression of muscle differentiation genes. qRT-PCR of muscle differentiation genes after treatment with FH535, XAV939, ß-catenin siRNA, or WNT3A for 48 h. GAPDH expression was used for normalization. Shown is the mean and SEM of three independent experiments measured in duplicates. MYOG, myogenin; DES, desmin; CHRNG, acetylcholine receptor gamma subunit; CKM, creatine kinase muscle. Significance level: ^*^p < 0.05.

Furthermore, the impact of WNT3A, FH535, XAV939, or ß-catenin siRNA on the differentiation status of the RMS tumor cell lines was analyzed by qRT-PCR. As seen in Figure 7 the β-catenin inhibitor XAV939 or siRNA mediated β-catenin knock down did not influence the expression of the muscle differentiation genes analyzed. Also, induction of canonical WNT pathway with WNT3A conditioned medium (Figure 7) as well as recombinant WNT3A (rWNT3A) in medium (Supplemental Figure 1) did not change the expression of the examined genes, whereas FH535 downregulated them in CRL-2061 (Figure 7). For the acetylcholine receptor this downregulation was confirmed on the protein level (Supplemental Figure 4).

To eliminate the influence of related WNT molecules that were secreted by the examined RMS tumor cells, all cell lines were also pretreated with either the porcupine inhibitor IWP2 (not shown) or LGK974 followed by stimulation with WNT3A conditioned medium or rWNT3A. The efficiency of the inhibitors was first tested with a WNT3A producing cell line (not shown). Pretreatment with LGK974 for 24 h and subsequent addition of WNT3A (either conditioned medium or rWNT3A) for 48 h induced *AXIN2* expression as described; however, it did not induce the myogenic program (Supplemental Figure 5).

### Long-Term Treatment With WNT3A Does Not Significantly Induce Differentiation or Attenuate “Rhabdosphere” Formation

**Figure 8:**
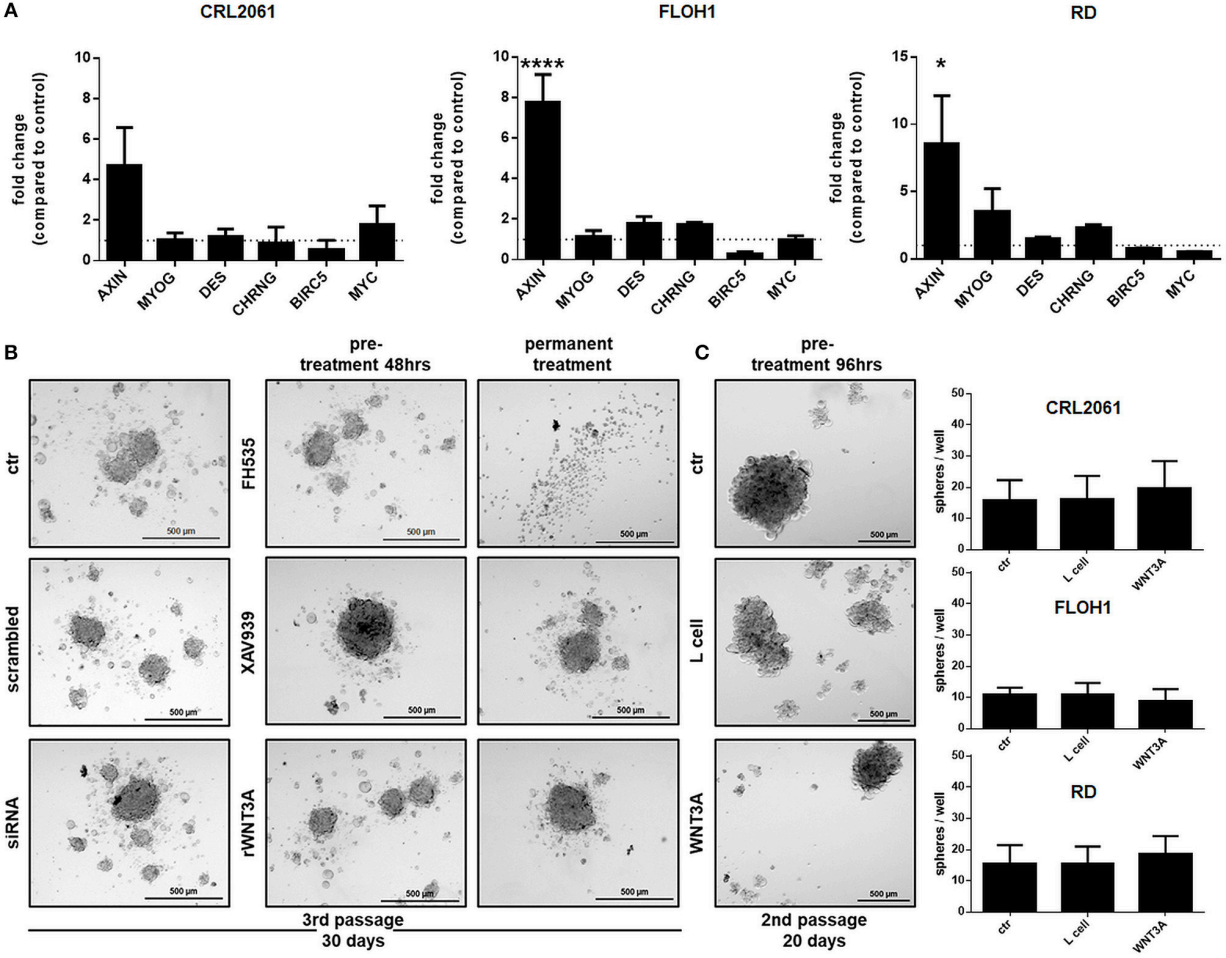
Effects of FH535, XAV939, ß-catenin-siRNA, or WNT3A on the expression of muscle differentiation markers and ß-catenin target genes, and on sphere formation ability. **(A)** qRT-PCR of muscle differentiation and ß-catenin target genes after treatment with WNT3A for 96 h. GAPDH expression was used for normalization. Shown is the mean and SEM of three independent experiments measured in duplicates. MYOG, myogenin; DES, desmin; CHRNG, acetylcholine receptor gamma subunit. **(B)** Pictures of sphere formation after pre-treatment with FH535 (20 μM), XAV939, ß-catenin-siRNA, or rWNT3A and with or without permanent FH535 (5 μM), XAV939 or rWNT3A treatment after 30 days or **(C)** pictures of sphere formation after a long term pre-treatment (96 h) with WNT3A. Right panel shows quantification of spheres after long term treatment with WNT3A. Shown are the mean values of three independent experiments performed in duplicates. Pictures were taken with a 50-fold magnification. Significance level: ^*^p < 0.05, ^****^p < 0.0001.

When cells were treated with WNT3A conditioned medium or rWNT3A up to 96 h, the expression pattern of myogenic differentiation markers and of *BIRC5* and *MYC* did not significantly change (Figure 8A and Supplemental Figure 1). In addition, we tested sphere formation ability of all three RMS tumor cell lines after a 48 h pretreatment with the inhibitors (due to toxicity of FH535) or a 96 h pretreatment with WNT3A (to induce muscle differentiation). Half of the sphere cultures were also treated permanently with the inhibitors or rWNT3A. The results show that sphere formation of all three RMS tumor cell lines was only inhibited by a 10-day permanent treatment with FH535, whereas no difference between control and treated cells was detected for the other substances (Figures 8B,C). However, the impact of FH535 on sphere formation was expected, since FH535 is a toxic substance for RMS tumor cell lines and induces apoptosis (please see also Figure 6).

### ß-Catenin Does Not Influence RMS Incidence or RMS Multiplicity in Mice

We finally evaluated the role of canonical WNT signaling in RMS growth in a genetic approach. For this purpose, we used heterozygous *Ptch*^*del*/+^ mutants that develop ERMS-like tumors. We first checked whether RMS of this model show active WNT signaling. For this purpose, we stained paraffin-embedded tissue sections for Bcl9, Bcl9-2, and for Lef1 that are all required for proper function of ß-catenin in the nucleus (31, 32). Indeed, the data show that in contrast to normal skeletal muscle, ß-catenin and Bcl9-2 are expressed in RMS. Bcl9 is expressed both in muscle and RMS whereas Lef1 is more present in RMS (Supplemental Figure 6). The data indicate that major components of Wnt signaling are present in the murine RMS.

**Figure 9:**
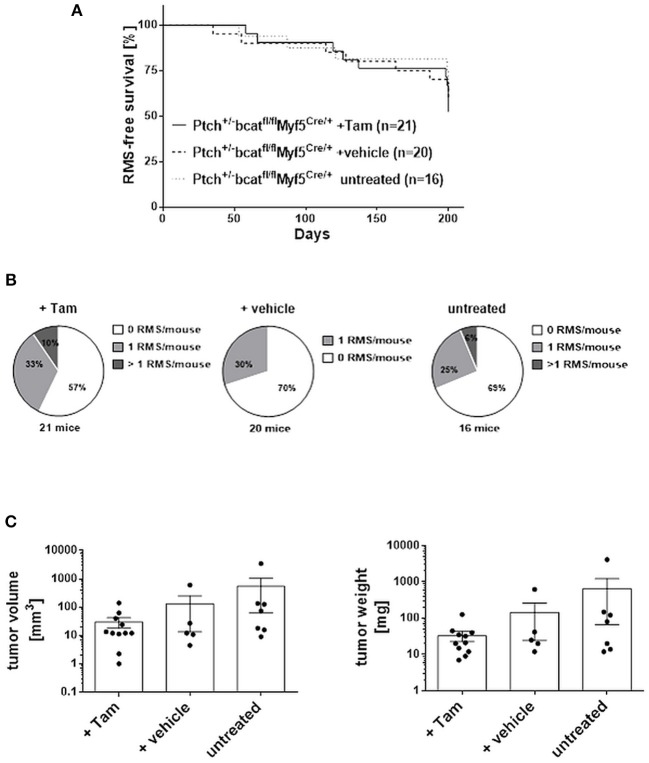
RMS incidence and multiplicity in ß*-cat*^*fl*/*fl*^*Ptch*^*del*/+^*Myf5*^*CreER*/*wt*^ animals. **(A)** Lifetime monitoring of RMS formation. According to Log-rank (Mantel-Cox) test and Gehan-Breslow-Wilcoxon test there were no significant differences. **(B)** Number of RMS per animal. Chi-square testing shows no significant differences. **(C)** Tumor volume and weight. One-Way ANOVA with Dunn's test for multiple comparisons shows no significant differences. Tam: tamoxifen-treated at an age of 4 weeks. Vehicle: vehicle-treated at an age of 4 weeks.

**Figure 10:**
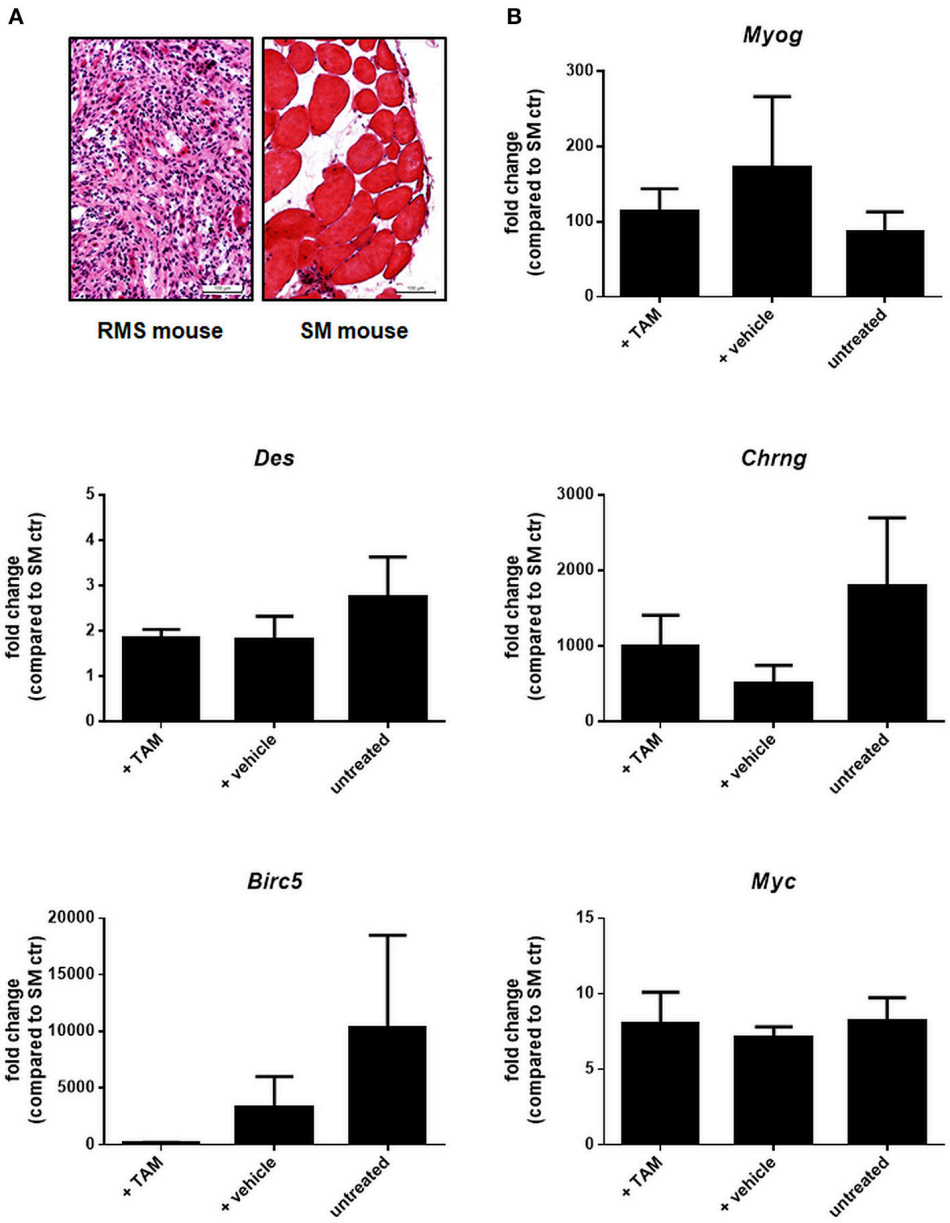
Expression of selected muscle differentiation markers and β-catenin target genes in ß*-cat*^*fl*/*fl*^*Ptch*^*del*/+^*Myf5*^*CreER*/*wt*^ animals. **(A)** H&E staining of a representative RMS and corresponding skeletal muscle (SM) of the same mouse to check tissue for tumor or skeletal muscle components before RNA isolation. **(B)** qRT-PCR of muscle differentiation markers and β-catenin target genes of RMS from untreated (*n* = 4), vehicle- (*n* = 5), or TAM-treated mice (*n* = 5). All data were normalized to GAPDH and are shown as fold change expression to the expression of the same gene within skeletal muscle (SM) of the respective mouse, which was set to 1. Bars show the mean and SEM of one cohort and represent measurements in technical duplicates. One-Way ANOVA with Dunn's test for multiple comparisons show no significant differences. Tam: tamoxifen-treated at an age of 4 weeks. Vehicle: vehicle-treated at an age of 4 weeks.

Next, *Ptch*^*del*/+^ mice were crossed with conditional β*-cat* mice and the respective mutation was set at an age of 4 weeks by induction of *Myf5Cre* using tamoxifen (Tam). Tam itself did not influenced overall RMS incidence or multiplicity (not shown). When the same parameters were analyzed in Tam-treated β*-cat*^*flox*/*flox*^*Ptch*^*del*/+^*Myf5*^*CreER*/*wt*^ mice and compared with the respective vehicle-treated or untreated control cohorts, there was also no significant difference (Figures 9A,B and Supplemental Table 2; for breeding scheme and recombination efficiency at the respective loci see Supplemental Figure 7). This shows that canonical WNT signaling apparently does not influence RMS incidence or multiplicity. Canonical WNT signaling also does not alter the growth rate of the tumor, because RMS weight and volume were also not different between the cohorts (Figure 9C). In addition, expression of the muscle differentiation markers *Myogenin, Desmin* as well as the *Chrng* or the β-catenin target genes *Birc5* and *Myc* were not affected by the conditional ß-catenin knock down (Figure 10).

## Discussion

It is known, that WNT signaling controls lineage commitment during embryogenesis (33). Since it is thought that childhood tumors are associated with a dysregulation of tissue development, it is comprehensible that WNT signaling plays an important role in initiation and maintenance of childhood tumors like Ewing sarcoma (34), osteosarcoma (35), retinoblastoma (36), neuroblastoma (37), medulloblastoma (38, 39), and pediatric ALL (40). This is apparently different in RMS, because neither specific β-catenin inhibition nor WNT3A-mediated stimulation of WNT/β-catenin signaling significantly influences cellular proliferation or differentiation of RMS cell lines in our experimental test setting. In addition, we also do not see any effect of a conditional β-catenin knockout on RMS incidence or RMS multiplicity in mice.

Independent oncogene mutation profiling's of pediatric tumors done by Chen et al., Shern et al., and Shukla et al., revealed that mutations in important components of the WNT signaling pathway, in particular β-catenin, are rare and only detectable in a subset of translocation negative RMS tumors(41–43). Moreover, immunohistochemical staining revealed that—with few exceptions in translocation negative tumors—RMS samples from patients express β-catenin exclusively in the cytoplasm of tumors cells (12, 13, 44). Therefore, it is tempting to speculate that RMS, with the exception of rare subtypes, do not belong to the group of tumors with WNT driven carcinogenesis. However, the importance of an active WNT signaling pathway for outcome and survival of RMS patients e.g., due to an activating mutation is not clear to date.

On the other hand, Annavarapu and colleagues described that WNT3A-driven activation of ARMS and ERMS cell lines through key components of the canonical WNT/β-catenin pathway had tumor suppressive function at least in ARMS. They showed that subsequent translocation of β-catenin to the nucleus induces expression of *MYOG, MYOD*, and *MYF5* and also reduces cell growth in the ARMS cell lines RH4 and RH30 but not the ERMS cell lines RD and RD18 (12). Similar observations have also been made by Singh et al. and Chen et al. who showed that activation of the canonical WNT signaling pathway either by LiCl or by using a GSK3 inhibitor increase the expression of muscle differentiation genes in ERMS (45, 46).

In contrast to these studies we now show that canonical WNT signaling and β-catenin apparently do not affect proliferation and myodifferentiation *in vitro* and in a murine RMS model *in vivo*. This conforms to our previous data showing that myodifferentiation of RMS is mainly dependent on LEF1 and TCF transcription factors and not on β-catenin activation or WNT3A (13, 21). The absence of any effect on RMS tumor cells in our current *in vivo* and *in vitro* knock down experiments indicate that β-catenin is of no importance for formation of RMS tumors. Our data is also in line with a recently published paper (14) reporting similar findings following another experimental approach. Bharathy et al., used an irreversible GSK3β inhibitor to achieve tonic canonical WNT signaling and elevated levels of β-catenin in human RMS samples. However, this had no effect on growth and myodifferentiation of patient-derived xenografts (14). Although the authors activated canonical WNT signaling with a GSK3β inhibitor, the results are identical to our results upon activation of canonical WNT signaling with conditioned WNT3A medium or rWNT3A. However, we here found that WNT3A slightly reduced the migration rate of RMS tumor cells. Since WNT3A in our setting does not affect ß-catenin, it is tempting to speculate that the reduced migration rate might be linked to an increased expression of cell adhesion molecules like N-cadherin or to some β-catenin-independent mechanisms (47–49).

In our setting, inhibition of canonical WNT signaling with XAV939, FH535, or ß-catenin-specific siRNA attenuated *AXIN2* expression. Nevertheless, neither XAV939 nor β-catenin siRNA had effects on proliferation and myodifferentiation or sphere formation ability *in vitro*. In contrast, FH535 strongly inhibited proliferation and differentiation of all RMS tumor cells and effectively induced apoptosis. These anti-proliferative and pro-apoptotic effects of FH535 have also been described for other tumor entities like colon carcinoma (50) and hepatocellular carcinoma (51). However, the potency of this β-catenin inhibitor depends on the structural similarity to the *PPARG* antagonist, GW9662 (15). It is therefore not surprising that FH535 treatment not only reduces β-catenin expression but also *PPARG* expression in RMS tumor cells. The different effects of FH535 compared to XAV939 and β-catenin siRNA could be explained by inhibition of PPARG rather than by the inhibition of β-catenin. Therefore, it is also conceivable that downregulation of differentiation markers (in RD and CRL2061) and the cell cycle arrest after FH535 treatment followed by apoptosis induction are rather mediated by *PPARG* in the RMS tumor cells than by β-catenin. Indeed, our unpublished data show that manipulation of the PPAR pathway with GW9662 induces apoptosis, inhibits migration and influences differentiation of RMS tumor cells in a similar manner as FH535 (unpublished results). In addition, an interdependency of PPARG and β-catenin has been described (52, 53). Thus, downregulation of PPARG is followed by a β-catenin upregulation and it was shown that deregulation of the balanced expression of PPARs and β-catenin can influence tumor growth, cell proliferation, cell invasiveness and angiogenesis (52, 54). However, the importance of the PPAR pathway for RMS tumors remains to be elucidated in the future.

The reasons for the discrepancies between our results, the studies of Annavarapu (12) or those of Singh et al., and Chen et al. (45, 46) in terms of β-catenin functions in RMS are not clear. As a matter of fact, each group used different cultivation conditions that are, however, very important for muscle differentiation e.g., the confluency of the cultures strongly influences the expression of muscle differentiation marker (55). In addition usage of different serum batches as well as the usage of bovine or horse serum affects the myogenic differentiation *in vitro*, too (56, 57). Therefore, a comparison of the studies is difficult. In addition, different pharmacological interventions were used: while Bharthany et al. used an inhibitor with high GSK3β specificity (14), Chen et al. (46) applied a less specific inhibitor that nowadays is known to block at least GSK3α in addition to the GSK3β isoform. However, Chen et al. reported an increased myodifferentiation, proliferation arrest and a reduced self-renewal capacity of ERMS, but not of ARMS, cell lines (46). In contrast, Annavarapu et al. who activated the canonical WNT pathway with rWNT3A described the opposite. They found ARMS as good responder cell lines, but not ERMS, although the same ERMS cell lines were used in experiments described by Chen et al. (46) and Annavarapu et al. (12). Finally, Chen et al. also described a reduced self-renewal capacity of ERMS tumor cells upon treatment with the GSK3 inhibitor and the following differentiation of the cells (46). However, we did not observe significant effects on sphere formation when treating the cells with WNT3A, XAV939, or β-catenin siRNA. This again support our results regarding myodifferentation, because sphere formation ability usually negatively correlates with the differentiation status of tumor cells (20, 46).

## Conclusion

*In vitro* the activation (or inhibition) of canonical WNT signaling using drugs or compounds that target the pathway at different levels can result in different outcomes, at least in RMS. Nevertheless, our results implicate that the major mediator of WNT signaling, β-catenin, only plays a subordinary role in growth and differentiation of RMS tumor cells. This is supported by our *in vivo* model. If the canonical WNT signaling pathway plays a role in differentiation and self-renewal capacity of RMS tumor cells, one would expect a higher RMS burden or faster growth of RMS in a β-catenin knockout mouse, which however, was not the case. However, the real role of β-catenin in RMS growth or differentiation most likely can only be assessed by analysis of patient data. It would be highly interesting to know the outcome of RMS patients with activating β-catenin mutations.

## Data Availability Statement

All relevant data is contained within the manuscript. All datasets for this study are included in the manuscript and the Supplementary Files.

## Author Contributions

NR, FV, and KS-K performed the *in vitro* experiments and were supported by MY. AS, NG, and JB performed the *in vivo* experiments, immunhistochemical staining and professional examination of the mouse tumors was done by H-US and FB. DB helped with the experiment design and manuscript concept. AM, HH, and KS-K are responsible for experiment design and wrote the manuscript.

### Conflict of Interest Statement

The authors declare that the research was conducted in the absence of any commercial or financial relationships that could be construed as a potential conflict of interest.

## Abbreviations

RMS: rhabdomyosarcoma
ARMS: alveolar rhabdomyosarcoma
ERMS: embryonal rhabdomyosarcoma.
