# Supplementary material for: Canonical WNT/β-Catenin Signaling Plays a Subordinate Role in Rhabdomyosarcomas
Source: Front Pediatr. 2018 Dec 5;6:378. doi: 10.3389/fped.2018.00378 (PMC6290061; doi:10.3389/fped.2018.00378)
Supplement: Supplementary file 1 [file Data_Sheet_1.PDF]

Supplementary Material

Canonical WNT/β-catenin signaling plays a subordinate role in rhabdomyosarcomas

Nada Ragab[1&], Florian Viehweger[1, 2&], Julia Bauer[3], Natalie Geyer[3], Mingya Yang[1], Anna Seils[1], Djeda Belharazem[1], Felix H. Brembeck[4], Hans-Ulrich Schildhaus[4], Alexander Marx[1], Heidi Hahn[3] and Katja Simon-Keller[1]*

***correspondence author:**

Dr. Katja Simon-Keller

katja.simon-keller@medma.uni-heidelberg.de

1 Supplementary Figures

1.1 Supplemental Figure 1

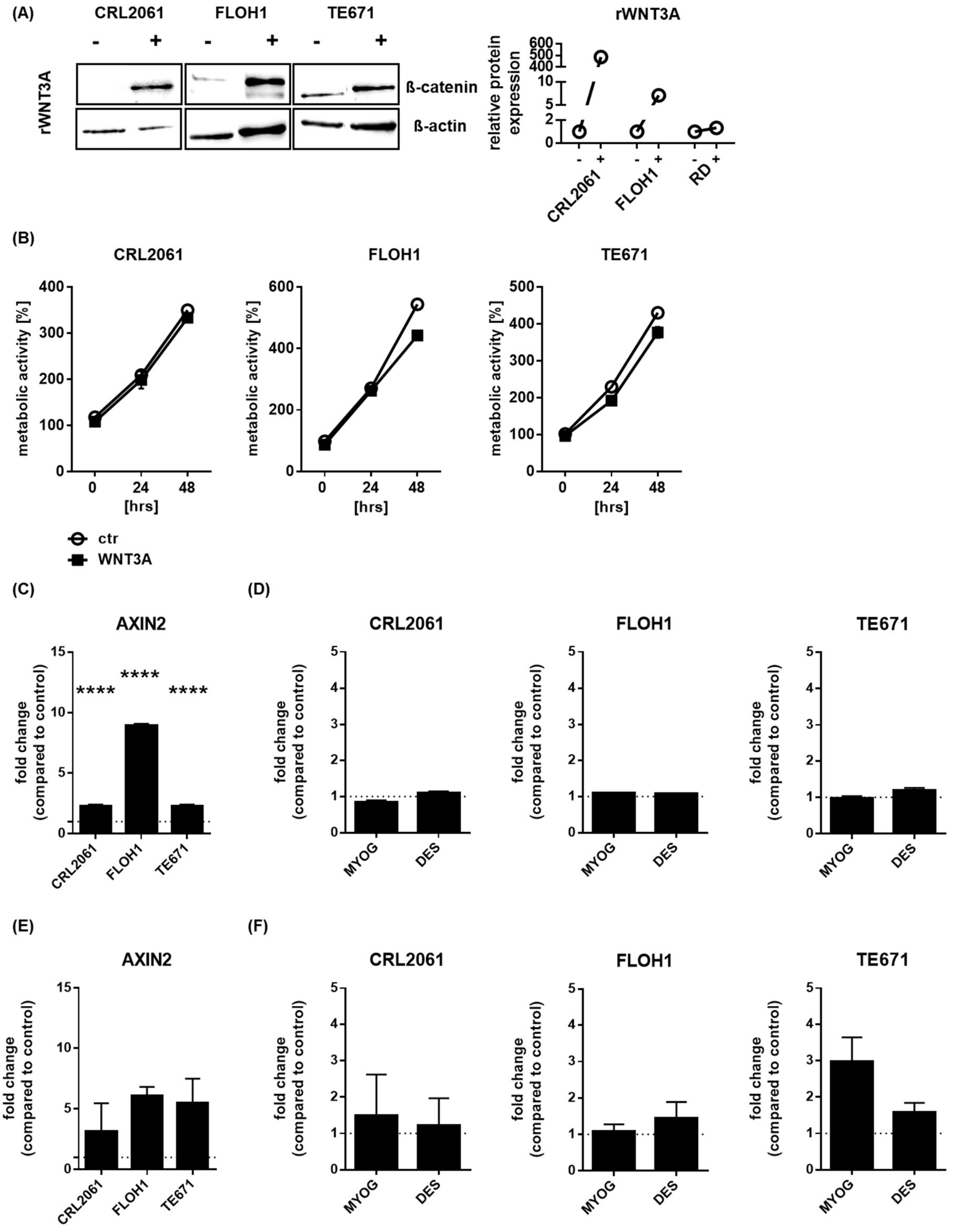

Supplemental Fig 1: Repetition of the experiments with recombinant WNT3A (A) Western Blot analysis of ß-Catenin expression in RMS tumor cell lines after rWNT3A treatment. ß-actin served as loading control. Right panel shows the corresponding densiometric analyisis (B) MTT test to examine the infuence of rWNT3A on CRL2061, FLOH1 and the ERMS cell line TE671. The graph show one representative experiment measured in triplicates (C) qRT-PCR of the ß-catenin target gene AXIN2 and (D) the muscle differentiation genes MYOGENIN and DESMIN after 48 h of rWNT3A treatment. (E) qRT-PCR of the ß-catenin target gene AXIN2 and (F) the muscle differentiation genes MYOGENIN and DESMIN after 96 h of rWNT3A treatment. GAPDH was used for normalization. Shown is the mean and SEM of two independent experiments measured in duplicates. Expression of untreated controls was set as 1.

1.2 Supplemental Figure 2

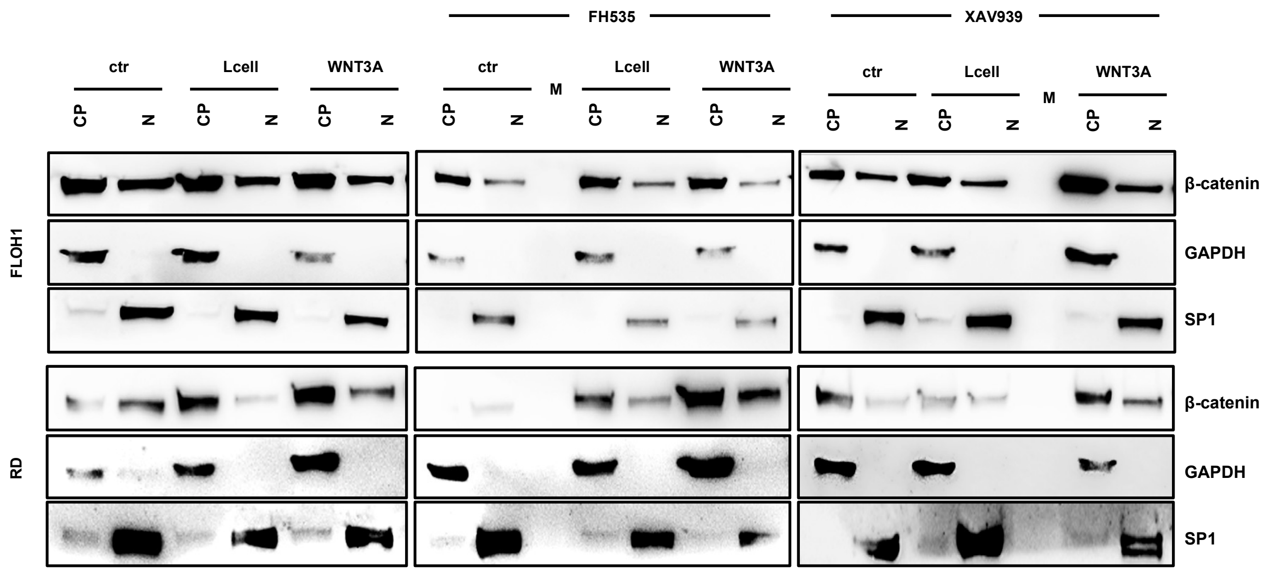

Supplemental Fig 2: Western Blot analysis of β-catenin expression in FLOH1 and RD tumor cells after FH535 or XAV939 treatment with or without WNT3A stimulation. Subcellular localization of ß-catenin is shown. GAPDH served as cytoplasmatic, SP1 as nuclear marker to check purity of the protein isolation from different compartments. CP – cytoplasmatic, N – nuclear.

1.3 Supplemental Figure 3

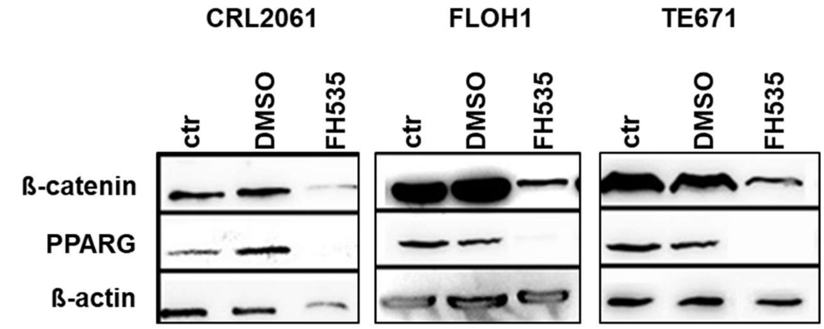

Supplemental Fig 3: Western Blot analysis of ß-catenin and PPARG expression in RMS tumor cell lines after FH535 treatment. ß-actin served as loading control.

1.4 Supplemental Figure 4

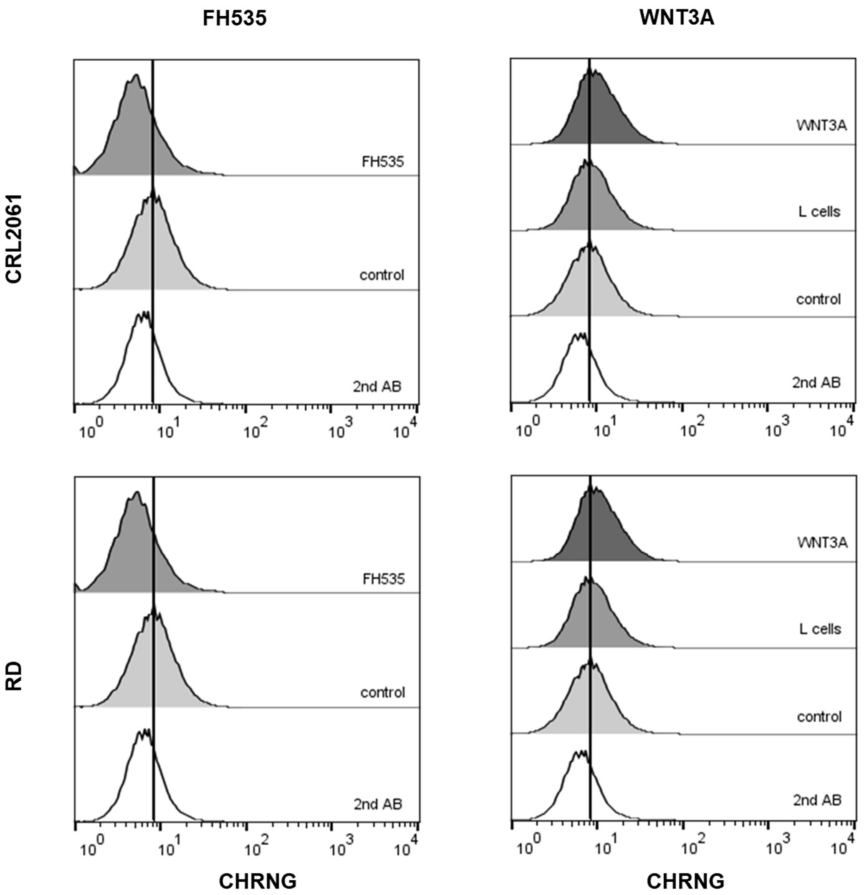

Supplemental Fig 4: Flow cytometry analysis of the RMS specific tumor antigen CHRNG after FH535 (left panel) and WNT3A (right panel) treatment. For a better discrimination the histogramms were shown on top of each other including an untreated control, a secondary antibody control and cells after treatment (dark grey). For WNT3A also the L-cell control is included.

1.5 Supplemental Figure 5

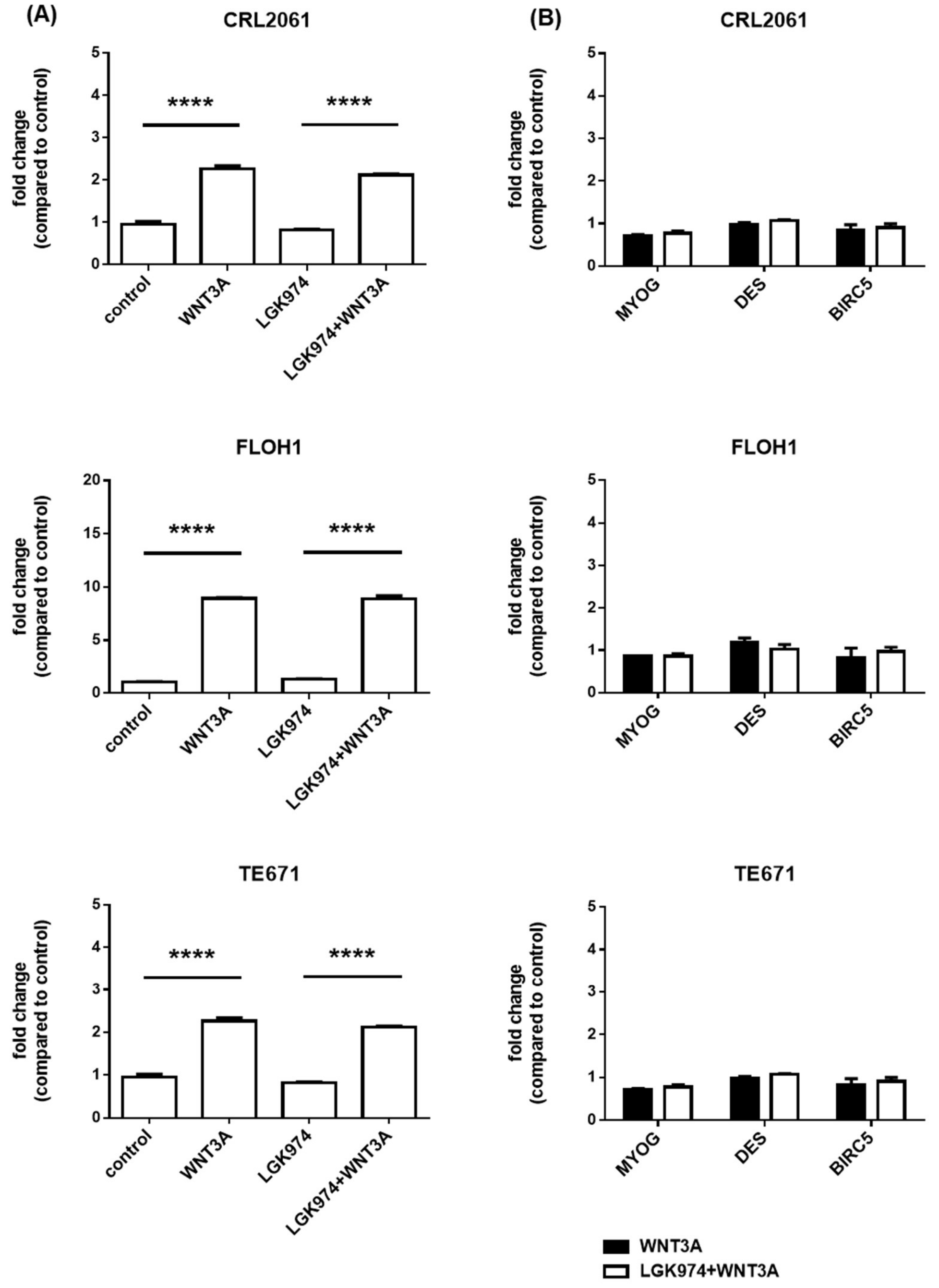

Supplemental Fig 5: Induction of the ß-catenin target gene AXIN2 after 24 h of rWNT3A treatment. Shown are qRT-PCR results without and after 24 hrs of pretreatment with the porcupine inhibitor LGK974 (B) expression of ß-catenin target genes after rWNT3A treatment without and with the porcupine inhibitor LGK974. GAPDH was used as reference. One representative experiment, measured in duplicates is shown.

1.6 Supplemental Figure 6

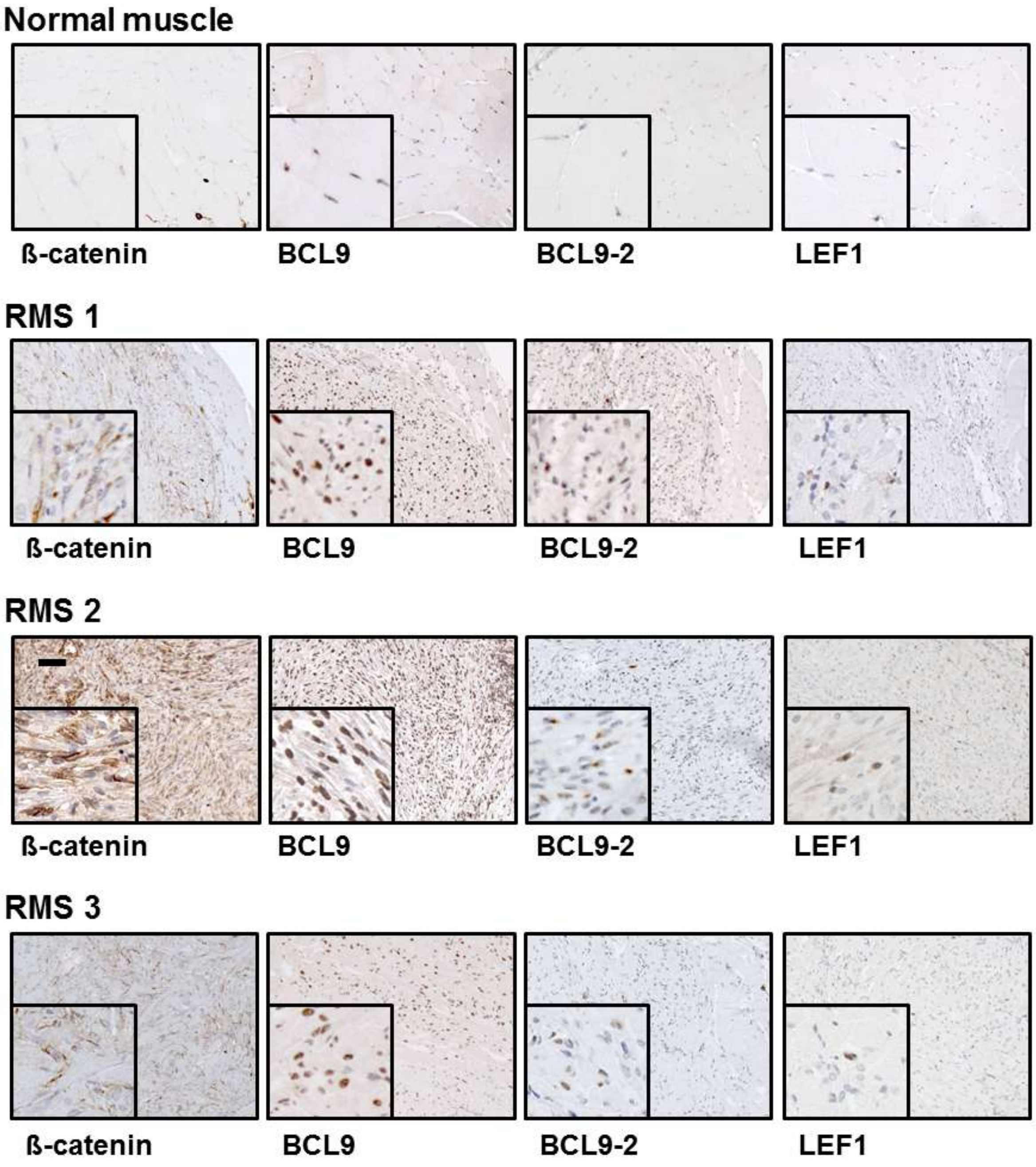

Supplemental Fig 6: Paraffin-embedded tissue stained for Bcl9, Bcl9-2 and for Lef1 that are all required for proper function of ß-catenin in the nucleus in normal muscle and three different RMS tissues.

1.7 Supplemental Figure 7

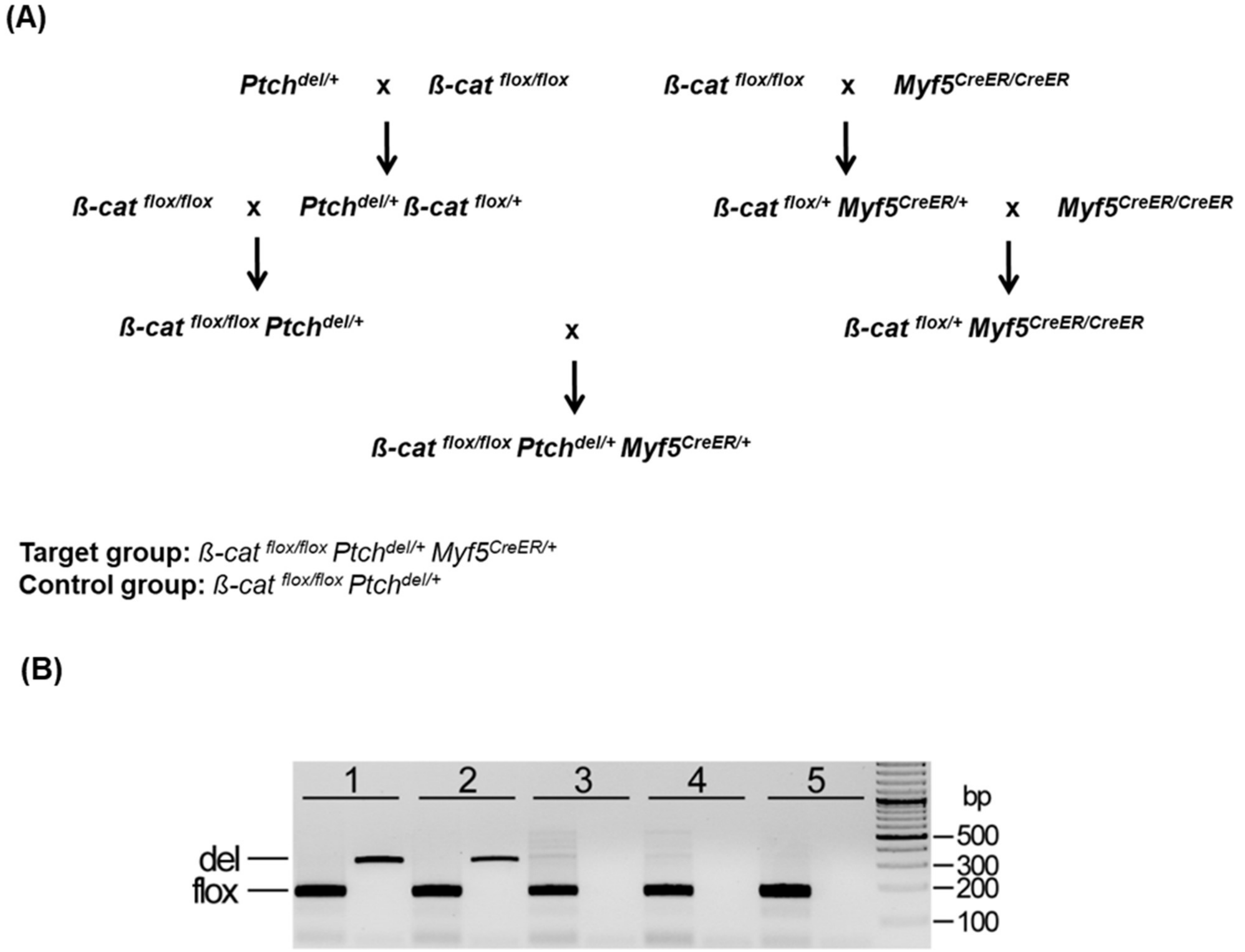

Supplemental Fig 7: (A) breeding sheme of the various mouse genotyps and (B) recombination at the floxed β-cat locus in RMS of β-catflox/floxPtchdel/+Myf5CreER/wt treated with tamoxifen or vehicle or remained untreated. Results show 5 RMS isolated from animals that have been treated with tamoxifen (1,2), vehicle (3,4) or left untreated (5). flox: floxed allele; del: deleted allele after treatment with tamoxifen. For primers see supplemental Table 1.

2 Supplementary Tables

Supplemental Table 1: Oligonucleotides used for PCR

qRT-PCR – Homo sapiens		
Primer Name	Primer Sequence (5’-3’ orientation)	
hAXIN2-F	GCCAACGACAGTGAGATATCC	exon 2 / exon 4
hAXIN2-R	CTCGAGATCAGCTCAGCTGCA	
BIRC5-F	AGCATTCGTCCGGTTGCGCT	exon 3 / exon 4
BIRC5-R	TCGATGGCACGGCGCACTTT	
CHRNG-F	GCGCTGGAGAAGCTAGAGAA	exon 10+11 / exon 12
CHRNG-R	CACCAGGAACCACTCCTCAT	
DESMIN-F	CATCGCGGCTAAGAACATTT	exon 4 / exon 5+6
DESMIN-R	GCCTCATCAGGGAATCGTTA	
CKM-F	GGAACTCTTTGACCCCATCA	exon 2 / 3
CKM-R	CTCCACCCTTGAGGTTTTCA	
MYC-F	GTAGTGGAAAACCAGCAGCC	Exon 1+2 / exon 2
MYC-R	CCTCCTCGTCGCAGTAGAAA	
MYOGENIN-F	CCAGGGGATCATCTGCTCAC	Exon 2+3 / 4
MYOGENIN-R	CTGTGATGCTGTCCACGATG	
**qRT-PCR – Mus musculus**		
Primer Name	Primer Sequence (5’-3’ orientation)	
Birc5-F	TGGCAGCTGTACCTCAAGAA	exon 1 / exon 2
Birc5-R	TCTATCGGGTTGTCATCGGG	
Chrng-F	ACGAAGGCCTGTGGATATTG	exon 4 / exon 5
Chrng-R	GAGAGCCACCTCGAAGACAC	
Desmin-F	CAAAGGGGTTCTGAAGTCCA	exon 8 / exon 8+9
Desmin-R	GTAGCCTCGCTGACAACCTC	
Myc-F	CCTTCTCTCCTTCCTCGGAC	exon 2 / exon 3
Myc-R	TGCCTCTTCTCCACAGACA	
Myogenin-F	CCAACCCAGGAGATCATTTG	exon 2+3 / 3
Myogenin-R	TCTGGGAAGGCAACAGACAT	
**genotyping PCR – mus musculus**		
Primer Name	Primer Sequence (5’-3’ orientation)	mouse line
exon7-F	AGGAAGTATATGCATTGGCAGGAG	$Ptch^{del}$ (950 bp)
Neo-R	GCATCAGAGCAGCCGATTGTCTG	
b-cat fwd	ACTGCCTTTGTTCTCTTCCCTTCTG	β-$catenin^{flox}$ b-cat fwd/b-cat rev: wt allele (143bp) and floxed allele (183bp) β-cat fwd/b-cat del-R5: deleted allele (332bp)
b-cat rev	CAGCCAAGGAGAGCAGGTGAGG	
b-cat del-R5	GAACAGTGCCATTCATCACTGAG	
cF	GCATTTCTGGGGATTGCTTA	Myf5CreER cF/cR: Cre allele (241 bp) CK 382/383: WT allele (454 bp)
cR	CCCGGCAAAACAGGTAGTTA	
CK382	ACCCTCCAGCTCCAGACTTATC	
CK383	CCCTGTAATGGATTCCAAGCTG	

Supplemental Table 2: Absolute numbers and percentages of RMS and other tumors of β-catflox/floxPtchdel/+Myf5CreER/wt animals with or without tamoxifen injection. The respective Kaplan-Meier curves are shown in Fig 9; LT – latentcy time; MB – medulloblastoma.

Genotype	n	Age, median [days]	All animals with RMS, median LT [days]	Animals with palpable RMS	Mice with other tumors or health problems	healthy/ unexplained death
β-cat$^{flox/flox}$Ptch$^{del/+}$ Myf5$^{CreER/wt}$ + Tam	21	148-211, 200	9 (43 %), 162	4 (19 %)	4 (1 cyst, 1 MB, 2 urinary retention)	10/0
β-cat$^{flox/flox}$Ptch$^{del/+}$ Myf5$^{CreER/wt}$ + vehicle	20	36-211, 183	6 (30 %), 121	5 (25 %)	8 (2 cyst, 4 MB, 2 urinary retention)	5/4
β-cat$^{flox/flox}$Ptch$^{del/+}$ Myf5$^{CreER/wt}$ untreated	16	105-202, 200	5 (31 %), 121	3 (19 %)	7 (1 cyst, 1 MB, 1 spindle cell tumor, 2 ulcer, 2 urinary retention)	3/3
